# Deutsche Gesellschaft für Radioonkologie (DEGRO) mahnt stationäre strahlentherapeutische Versorgung an: Stellungnahme zu einem Urteil des Bundessozialgerichts vom 29.08.2023 zur Erbringung stationärer strahlentherapeutischer Leistungen

**DOI:** 10.1007/s00066-023-02169-4

**Published:** 2023-10-23

**Authors:** Rainer Fietkau, Ulrike Höller, Michael van Kampen, Mechthild Krause, Cordula Petersen, Dirk Vordermark

**Affiliations:** 1 Strahlenklinik, Friedrich-Alexander-Universität, Erlangen-Nürnberg, Erlangen, Deutschland; 2 Deutsche Gesellschaft für Radioonkologie DEGRO, Berlin, Deutschland; 3 Radioonkologische Klinik, Krankenhaus Nordwest, Frankfurt/Main, Deutschland; 4 Klinik und Poliklinik für Strahlentherapie und Radioonkologie, Universitätsklinikum Carl Gustav Carus, Technische Universität Dresden, Dresden, Deutschland; 5 Klinik für Strahlentherapie und Radioonkologie, Universitätsklinikum Hamburg-Eppendorf, Martinistr. 52, 20246 Hamburg, Deutschland; 6 Klinik für Strahlentherapie, Universitätsklinikum Halle (Saale), Halle (Saale), Deutschland

Das Bundessozialgericht hat mit Urteil vom 29.08.2023 festgestellt, dass ein Krankenhaus, das keinen Versorgungsauftrag für die Erbringung strahlentherapeutischer Leistungen hat, solche Leistungen stationär nicht erbringen und abrechnen kann (Verhandlung B 1 KR 18/22 R).

Die Strahlentherapie ist eine wesentliche Säule der Behandlungskonzepte von Tumorerkrankungen. Sie wird in Deutschland überwiegend ambulant erbracht. Patient:innen mit starker Symptombelastung durch die Tumorerkrankung, mit starken therapiebedingten Nebenwirkungen oder mit sehr intensiven und belastenden Therapiekonzepten – z. B. einer kombinierten Radiochemotherapie – benötigen jedoch eine stationäre Betreuung während der Strahlenbehandlung. Dabei kann der stationäre Behandlungsbedarf bereits ab Beginn einer Strahlenbehandlung bestehen oder erst im Verlauf einer ambulant begonnenen Bestrahlungsserie auftreten. Diese stationäre Aufnahme ist bei bestimmten Diagnosen sogar unabdingbar.

In Deutschland werden kontinuierlich pro Jahr etwa 90.000 stationäre Behandlungsfälle mit in Summe etwa 350.000 Strahlentherapieleistungen erbracht [1]. Die aktuelle Statistik des Bundesamtes für Strahlenschutz kommt zu dem Ergebnis, dass pro Jahr etwa 43.000 Strahlentherapien von Tumorerkrankungen zumindest teilweise stationär durchgeführt wurden, wobei berücksichtigt ist, dass innerhalb einer Strahlentherapie mehrere stationäre Aufenthalte („Fälle“) erforderlich werden können [2]. Nach diesen Zahlen werden knapp 20 % der Strahlentherapien von Tumorerkrankungen stationär erbracht.

Die Erbringung strahlentherapeutischer Leistungen in Deutschland beruht bisher auf verschiedenen organisatorischen Modellen. Neben Krankenhäusern mit eigener Klinik oder Abteilung für Strahlentherapie mit häufig auch eigenem stationärem Bereich haben sich an vielen Standorten Kooperationsmodelle mit Erbringung strahlentherapeutischer Leistungen ausschließlich durch einen Kooperationspartner (Praxis oder Medizinisches Versorgungszentrum) entwickelt.

Die Deutsche Gesellschaft für Radioonkologie (DEGRO) sieht in der mit dem o. g. Urteil bestehenden Rechtslage zusammen mit den über die letzten Jahre geschaffenen Strukturen einer teilweise kompletten Trennung der ambulanten von der stationären Versorgung (Abschaffung der Strahlentherapie-Hauptabteilungen an Krankenhäusern) eine massive Gefährdung der Versorgung von Tumorpatient:innen in Deutschland. In den derzeitigen Versorgungsstrukturen verfügen viele Krankenhäuser nicht (mehr) über einen stationären Versorgungsauftrag für die Erbringung strahlentherapeutischer Leistungen und können somit derzeit keine stationäre Versorgung strahlentherapeutischer Patient:innen übernehmen.

Die DEGRO geht davon aus, dass aufgrund der weiten Verbreitung der genannten Kooperationsmodelle die vorhandenen Kapazitäten zur Erbringung stationärer Strahlentherapieleistungen an Kliniken mit entsprechendem Versorgungsauftrag derzeit in einigen Regionen nicht ausreichen, um den Bedarf an stationärer Strahlentherapie abzudecken. Aus medizinischer Sicht ist es essenziell, dass Versorgungsstrukturen rechtskonform so weiterentwickelt werden, dass Indikationen zur Strahlentherapie weiterhin allein anhand medizinischer Kriterien gestellt werden können und die in der Regel mehrwöchige Therapie zeitgerecht und ohne Unterbrechung durchgeführt werden kann, auch dann, wenn sie eine stationäre Behandlung erfordert.

Es besteht vor dem Hintergrund des aktuellen Urteils des Bundessozialgerichts eine dringende Notwendigkeit, Anpassungen zur Sicherstellung der Versorgung vorzunehmen. Zu betrachten sind strukturelle Änderungen mit Stärkung der stationären Strahlentherapie an den Klinikstandorten, die eine Erbringung stationärer Strahlentherapieleistungen auch dann ermöglichen, wenn ein Patient/eine Patientin unerwartet stationär behandelt werden muss. Die Absicherung der stationären strahlentherapeutischen Behandlung von Tumorpatient:innen muss in der aktuellen Planung zur Krankenhausreform berücksichtigt werden als eigener Leistungsbereich Strahlentherapie.
